# Radiomics Based Bayesian Inversion Method for Prediction of Cancer and Pathological Stage

**DOI:** 10.1109/JTEHM.2021.3108390

**Published:** 2021-08-30

**Authors:** Hina Shakir, Tariq Khan, Haroon Rasheed, Yiming Deng

**Affiliations:** 1 Department of Electrical EngineeringBahria University 66736 Karachi 75620 Pakistan; 2 Department of Electrical and Power EngineeringNational University of Science and Technology 108243 Islamabad 75350 Pakistan; 3 Department of Electrical and Computer EngineeringMichigan State University 3078 East Lansing MI 48824 USA

**Keywords:** Bayesian inversion, cancer stage estimation, nodule classification, particle filter, radiomic features

## Abstract

Objective: To develop a Bayesian inversion framework on longitudinal chest CT scans which can perform efficient multi-class classification of lung cancer. Methods: While the unavailability of large number of training medical images impedes the performance of lung cancer classifiers, the purpose built deep networks have not performed well in multi-class classification. The presented framework employs particle filtering approach to address the non-linear behaviour of radiomic features towards benign and cancerous (stages I, II, III, IV) nodules and performs efficient multi-class classification (benign, early stage cancer, advanced stage cancer) in terms of posterior probability function. A joint likelihood function incorporating diagnostic radiomic features is formulated which can compute likelihood of cancer and its pathological stage. The proposed research study also investigates and validates diagnostic features to discriminate accurately between early stage (I, II) and advanced stage (III, IV) cancer. Results: The proposed stochastic framework achieved 86% accuracy on the benchmark database which is better than the other prominent cancer detection methods. Conclusion: The presented classification framework can aid radiologists in accurate interpretation of lung CT images at an early stage and can lead to timely medical treatment of cancer patients.

## I. Introduction

Image analysis techniques have successfully provided personalized prognosis and treatment plans for cancer patients with a greater accuracy in the recent years. There are several studies available that show the importance of imaging features for treatment, monitoring and outcome prediction in lung cancer as well as other cancer types [1], [2]. Besides prognosis, the quantitative imaging features also known as radiomic features have been extracted from the medical images to be further used in clinical research for lung cancer diagnosis [3], [4]. Most of the prominent radiomics based classification methods for lung cancer have incorporated neural networks and deep learning approaches [5]–[13]. A multi-view convolutional network is proposed in [9] for binary (benign and malignant) and ternary classification (binary, primary malignant and metastatic malignant). The proposed model achieved error rate of 5.41% for binary classification and 13.91% for ternary classification respectively. Another multi-view knowledge-based collaborative (MV-KBC) deep model classified nodules on limited chest CT data in [11]. The prediction error computed in terms of Root Mean Square Error (RMSE) of the model is 0.62. Authors in [12] investigated the usefulness of a deep convolutional neural network (DCNN) for benign, primary lung cancer and metastatic cancer classification. The best averaged accuracy of 68% was achieved with transfer learning for image size of 224.

Another common approach adopted to address classification problem is the application of machine learning classifiers. A structural co-occurrence matrix (SCM) based method was proposed by [13]. The proposed technique did not only classify the nodules as benign or malignant but also classified the malignant nodules into 5 prescribed levels with an accuracy of 74.5%. Yu *et al.* in [5] presented a study to detect pathological stages I, II, III and IV respectively in non-small cell lung cancer (NSCLC) using Random Forest algorithm. Out of the bag (OOB) error was computed to measure classification accuracy of the Random Forest algorithm whereas the predicted cancer stages of test data sets achieved ROCs between 0.6 to 1.

However, there is a dearth of scientific approaches to diagnose the benign or cancerous nature of a lung nodule along with its pathological stage in case of malignancy. Furthermore, the insufficient size of training data sets required for neural networks training is a challenge for accurate cancer stage detection of the tumor. Keeping in view the aforementioned challenges, there is an emerging need to investigate new sophisticated solutions for the detection of malignancy status in lung nodules. A useful approach towards joint estimation of tumor malignancy and its stage can involve tracking of tumor characteristics using temporal analysis of CT images [14], [15]. Such an approach also offers good potential to address the problem of smaller data sets in medical imaging.

In this research article, a Bayesian inversion method is proposed which employs longitudinal radiomics data extracted from CT scans of lung nodules to detect cancer and its pathological stage. The presented system adopts particle filtering approach to compute the posterior Probability Density Function (PDF) of cancer occurrence and cancer pathological stage respectively. For PDF estimation, a joint likelihood function incorporating diagnostic radiomic features is developed which can diagnose malignant nodules and the pathological cancer stage. The cancer stage is determined as early stage cancer (stage I & stage II) or advanced stage cancer (stage III & stage IV) respectively. Furthermore, radiomic features which can discriminate between cancer stages are also investigated and evaluated.

The presented Bayesian inversion method was validated on a total of 200 nodules with 130 malignant and 70 benign nodules. There were 109 malignant and 63 benign nodules which were successfully diagnosed showing better performance of the proposed framework than the other prominent methods in literature.

In the following sections, research problem is formulated and proposed stochastic frame work is described. Next, implementation of the presented model is discussed. Then, results and discussion are presented followed by a conclusion. The notations used in this research work are listed in Table 1 for reader’s understanding.

**TABLE 1:** List of Notations

Annotations	Description
$k$	Screening time in quarters
$U$	Set of diagnostic scores as states
$U_{k}$	Diagnostic score (state) at time $k$
$K$	Total number of quarters
$Z_{k}$	Set of diagnostic radiomic feature measurements
$z_{qk}$	$q^{th}$ diagnostic feature measurement at time $k$
$N_{s}$	Total number of samples
$u_{i}^{k} $	$i^ {th}$ particle at screening time $k$
$w_{i}^{k} $	Weight of $i^ {th}$ particle at screening time $k$
$p$	$m^{th}$ coefficient of measurement function
$M$	Order of measurement function
$\xi $	Predefined convergence threshold
$N$	Number of iterations

## II. Problem Formulation

A number of radiomic features depicting shape and gray level intensities of lung nodule have shown diagnostic power towards lung, colon and head and neck cancer [16]. The aim of the presented research is to utilize such radiomics features measurements from periodic chest screening images in a stochastic framework to detect lung cancer. The sequential evaluation of nodules’ malignancy status given the radiomics data from periodic screening images is an inverse problem and can be solved using Bayesian inversion approach [17]. There are several methods available to solve the aforesaid problem but a recursive Bayesian filter based on sequential Monte Carlo method is proposed since it uses nonlinear models [18]. The need of nonlinear modeling arises due to the nonlinear behavior of measured radiomics features and the associated likelihood function for lung cancer diagnosis [16].

**FIGURE 1.**
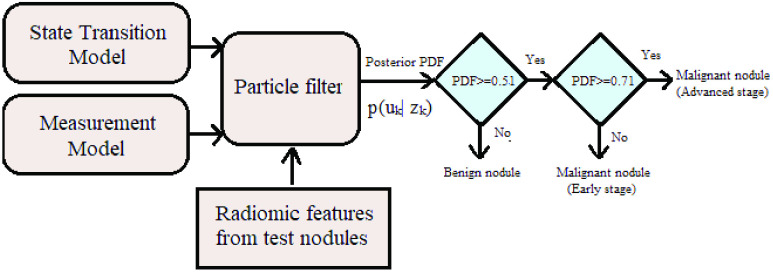
Work flow of the Bayesian inversion method implemented in proposed system for multi-class classification of lung nodules.

**FIGURE 2.**
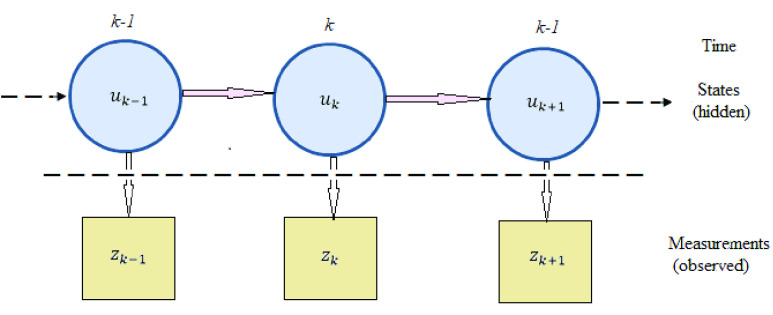
Illustration of proposed inverse problem with hidden states and their observed measurements.

The proposed Bayesian inversion framework requires a likelihood function for PDF estimation. Since multi-class classification using a single mathematical model has not been a preferred approach due to the complex relationship between benign and cancerous (multiple stages) nodules [19], [20], an intuitive approach has been adopted to combine two mathematical likelihood equations into one for PDF estimation. The proposed system for cancer prediction is shown in Fig. 1. In practice, an inverse problem can be represented by two models namely state transition model and measurement model [21] and is shown in Fig. 2 with states represented by $u$ and measurements by $z$. A description of these two models and their application in Bayesian inversion method for malignancy estimation is given in the following section.

## III. Diagnostic Stochastic Framework

### A. Stochastic State-Space Models

To start with, a state vector $\mathit {U \!=\! \{ u_{1},\! u_{2},\! u_{3},\! u_{4},.\ldots.,u_{k-1}, u_{k}\} }$ is defined where each element $u_{k}$ is a diagnostic score between 0 and 1 that represents the benign/malignancy state of a lung tumor at a screening interval $k$. A state transition model represents the evolution of state $u_{k}$ with respect to time instants and can be expressed as [22]:

\begin{equation*} u_{k} = x_{k-1} (u_{k-1}, v_{k-1})\tag{1}\end{equation*}

Here $x_{k}(.)$ is a non-linear and time varying function that models the state transition process and $v_{k}$ is process noise respectively.

The $q$ number of diagnostic radiomic feature measurements of a lung nodule in a CT image at each screening interval $k$ are assumed to be $z_{1k},z_{2k},\ldots,z_{qk}$ and are represented by a measurement vector $Z$ such that $\mathit {Z\!=\! \{z_{11},z_{21},\ldots.,z_{q1},z_{21},z_{22},\ldots z_{q2},\!\ldots,z_{1k},z_{2k},\ldots,z_{qk}\}}$. The measurement function relates the features measurements to the true malignancy states of a lung tumor and can be expressed as:

\begin{equation*} z_{k}=y_{k} (u_{k}, n_{k})\tag{2}\end{equation*}

Here $y_{k}(.)$ is a time varying and independent function that represents measurement model and $n_{k}$ is the measurement noise. Since, there has been a lack of discussion on the linearity of radiomics based state transition and measurement functions in the literature, a more generalized approach is adopted to perform the Bayesian inversion estimation which is offered by particle filter to estimate the posterior probability of malignancy and pathological cancer stage respectively in lung nodules.

### B. Particle Filtering

A particle filter is an algorithm which represents the desired posterior PDF of an unknown state as weighted samples, also known as particles. Using a large number of samples (particles), the estimated PDF is approximated to the true PDF. In particle filtering approach to dynamic state estimation of a lung nodule, the posterior PDF of the unknown malignancy state $u_{k}$ is constructed based on the set of received feature measurements $Z_{k}$.

Given the likelihood $p(Z_{k}|u_{k})$ of measured diagnostic features $Z_{k}$ at time $k$, the posterior density $p(u_{k}|Z_{k})$ of unknown state $u_{k}$ for the set of received features $Z_{k}$ can be computed using Bayes’ Theorem [23] as follows:

\begin{equation*} p(u_{k} \mid Z_{k})=\frac {p(Z_{k} \mid u_{k})p(u_{k} \mid Z_{k-1})}{p(z_{k} \mid Z_{k-1})}\tag{3}\end{equation*}

Here the normalizing constant $p(z_{k} \mid Z_{k-1})$ is given by:

\begin{equation*} p(z_{k} \mid Z_{k-1})=\int p(z_{k} \mid U_{k})p(U_{k} \mid Z_{k-1})du_{k}\tag{4}\end{equation*}

The normalization constant depends upon the likelihood function $p(Z_{k}|u_{k})$, defined by the measurement model in Eq. (2). Recursively, the filter predicts and updates to estimate the sequential unknown malignancy states of nodules. In summary, the proposed particle filter expresses the PDF $p(u_{k}|Z_{k})$ of a nodule’s unknown state at screening interval $k$ as a set of weighted samples. The total number of samples drawn from an importance density are assumed $N_{s}$. We represent a sample point as $u_{k}^{i}$ and its associated weight as $w_{k}^{i}$ where $\mathit {i= \{1, 2,\ldots., N_{s} \}}$. A randomly measured weighted sample at any screening interval can be represented as a set $\{u_{k}^{i},w_{k}^{i}\}$. Using the principle of importance sampling, the weights are normalized such that ${\sum {w_{k}^{i}}=1}$. Then, the posterior density at a screening time $k$ can be approximated as:

\begin{equation*} p(u_{k} \mid Z_{k}) = \sum _{i=1}^{N_{s}} w_{k}^{i} \delta (u_{k}-u_{k}^{i})\tag{5}\end{equation*}

The samples can be drawn from the importance density $q(Z_{k}^{i}|U_{k})$ and the weights can be assigned using the following association:

\begin{equation*} w_{k}^{i} \propto \frac {p(Z_{k}^{i} \mid U_{k})}{q(Z_{k}^{i} \mid U_{k})}\tag{6}\end{equation*}

If the samples at screening time $k-1$ are available constituting to probability $p(z_{1:k-1}\mid u_{1:k-1})$, then $p(z_{1:k}\mid u_{1:k})$ is approximated with a new set of samples once the observation $z_{k}$ is known at the nodule screening time $k$.

The prior or state transition probability $p(u_{k}|u_{k-1})$ of malignancy in a nodule is modeled using exponential distribution because the progression rate of lung cancer follows exponential distribution [24]. Since the transition density depends upon the malignancy states of cancer $u_{k-1}$ and $u_{k}$ at any consecutive times $k-1$ and $k$ respectively, it can be represented as follows:

\begin{equation*} p(u_{k} \mid u_{k-1})=e^{- \frac {{\mid u_{k}-u_{k-1}\mid }^\alpha }{\alpha }}\tag{7}\end{equation*}

where $\alpha $ is a scalar value that controls the variability in the predicted state value.

## IV. Implementation of Particle Filter

The test datasets which are given as input to the proposed model comprises of longitudinal radiomics data of patients’ CT images acquired from National Lung Screening Trial (NLST) [25].

### A. NLST Datasets

NLST is a randomized multi-site trial which enrolled 53,454 people with high cancer risk to assess the impact of using low dose CT screening in mortality reduction from lung cancer. In NLST project, low dose CT screenings of the enrolled people were performed for three consecutive years which are termed as T0, T1 and T2 respectively. For the implementation of proposed filter, longitudinal data of participants who had cancer detected after the third year (T2) of screening were included. This ensured availability of CT data for all the three years in order to apply the prediction model. A total of 130 cases with positive screening at either year T0 or T1 followed by screen detected cancer at year T2 were acquired. Another 70 CT data sets with benign nodules detected at year T2 and with positive screening at either year T0 or T1 were accessed from the database.

The detected nodules from all the 3 consecutive CT screenings of each patient were segmented and 105 3-D features were computed. This resulted in a set of 3 longitudinal radiomic readings for each patient. In order to tackle the constraint of fewer data points, single data point was interpolated using cubic-spline method [17] between data obtained from year T0 and year T1, and between year T1 and year T2 respectively. The total number of screening intervals after interpolation were 5($K =5$) within a duration of 3 years. Starting with an initial CT scan, there are 4 sample points following and each point represents a half-yearly CT screening data of the patient which was fed into the proposed system for PDF estimation.

### B. Likelihood Function for Cancer and Cancer Stage Detection

For PDF estimation using particle filter, a likelihood function has to be developed. In the literature reviewed, authors in [16] formulated a likelihood function for cancer detection (benign or malign) incorporating two highly diagnostic radiomic features namely Surface volume ratio and Sum entropy values of a nodule and is given as:

\begin{align*} p_{1}(Z_{k}|u_{k}) \!=\! a+b(z_{1k})+c(z_{1k}^{2})\!+\!d(z_{1k}^{3})+e(z_{1k}^{4})+fln(z_{2k}) \\\tag{8}\end{align*}

Here $z_{1k}$ represents the Surface volume ratio and $z_{2k}$ denotes the Sum entropy value of nodule at $k^{th}$ quarter. The value of $p_{1}(Z_{k}|u_{k})$ is below 0.51 for benign state and equal or above 0.51 for malignancy in the nodule. The coefficients of Eq. (8) assume the following values [16]:

\begin{align*} a=&0.7478;~b = 2.2268;~c = - 5.5856;\\ d=&3.6318;~e = -0.73065;~f = 1.2814E-02\end{align*}

As reported in a research study [26], a single multi-class likelihood function is prone to large errors hence cancer detection and cancer stage detection have to be performed using two distinct mathematical functions.

**FIGURE 3.**
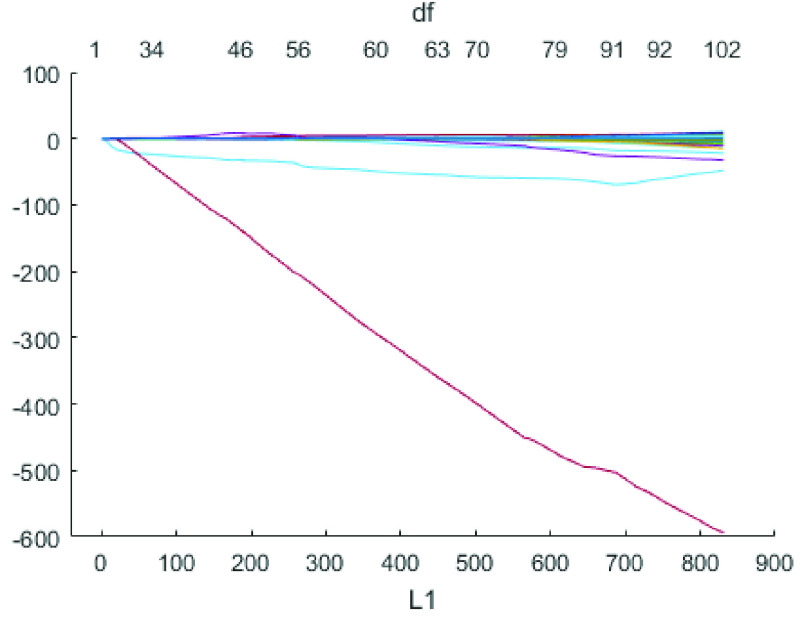
LASSO plot of coefficients fit.

Malignancy with cancer pathological stage I or II was collectively termed as early stage cancer whereas stage III and IV cancer was termed as advanced stage cancer [27]. The training cohort comprised of 73 nodules with early stage cancer and 127 nodules with advanced stage cancer from Lung1 [28] database. A pre-processing step of features extraction and features reliability test [16] was applied to the training data sets which resulted in 51 stable and reliable features reported in Table SI (Supplementary material). Furthermore, the redundant features were removed and highly diagnostic features were selected using Least absolute shrinkage and selection operator (LASSO) [10], a method of regression analysis. LASSO technique performs L1 regularization of the features via penalized estimation function to achieve a reduced set of diagnostic features which were validated using 5-fold cross validation. Fig. 3 shows the LASSO coefficients of predictors on y-axis and the L1 norm (the penalization parameter also known as lambda) of coefficients on x-axis for a regression fit. The top x-axis of the plot represents degree of freedom denoted by df and it shows the non-zero coefficients of predictors. Following 7 diagnostic features were obtained after the application of LASSO technique (lambda = 0.0282 with minimum MSE) on pre-processed features:

1) Sphericity
2) Dependence Variance (DV)
3) Large Dependence High Gray Level Emphasis (LDHGLE)
4) Cluster Prominence (CP)
5) Large Area Low Gray Level Emphasis (LALGLE)
6) Small Area Emphasis (SAE)
7) Strength

The LASSO coefficients of the aforementioned diagnostic features are reported in Table SII. Their diagnostic power ranking was computed using mean scores of 3 supervised feature ranking approaches namely feature based Neighborhood Component Analysis (fNCA), ReliefF network and Infinite Latent Feature Selection (ILFS) respectively [29]. Out of these 7 coefficients, five most discriminating features were used to formulate the likelihood function for early stage or advanced stage cancer detection as follows:

\begin{align*}&\hspace {-2pc} p_{2}(Z_{k} \mid u_{k}) \\=&1.2 -1.1348(z_{3k}) -7.4597e^{-07}(z_{4k}) \\&+ 3.0780e^{-08}(z_{5k}) +0.3917(z_{6k}) -0.00361(z_{7k})\tag{9}\end{align*}

**FIGURE 4.**
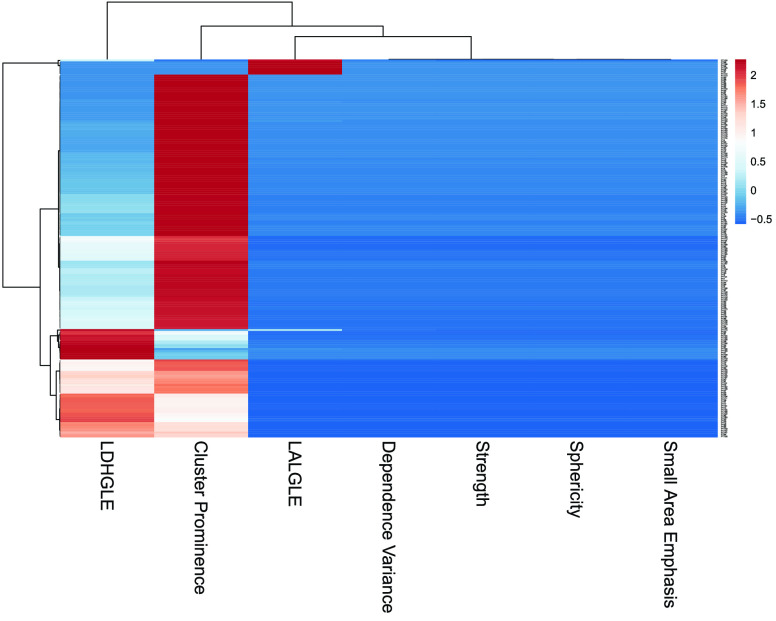
Hierarchical clustering of selected radiomic features as a heatmap.

Here $p_{2}(Z_{k}|u_{k})$ indicates likelihood of cancer stage as early stage or advanced stage cancer. It produces a value between 0.51 and below 0.70 for early stage cancer and assumes a value between 0.70 and 0.90 for the detection of advanced stage cancer. The features $z_{3k}$, $z_{4k}$, $z_{5k}$, $z_{6k}$ and $z_{7k}$ represent Sphericity, LDHGLE, Cluster Prominence, Small Area Emphasis and Strength respectively for $k^{th}$ CT screening. The coefficients $z_{4k}$, $z_{5k}$ of features LDHGLE and Cluster Prominence respectively are very small and can be excluded. The proposed likelihood function was validated on 47 nodules with early stage cancer and 143 nodules with advanced stage cancer. The function detected 40 nodules with early stage cancer and 125 nodules with advance stage cancer following the designated score ranges. Overall, the proposed function achieved an accuracy of 86.84% (CI:81.19% to 91.30%), specificity of 85.11% (CI:71.69% to 93.80%) and sensitivity of 87.41% (CI:80.84% to 92.37%) respectively with an AUCROC of 84.61%. The ROC plot of the nodule pathological stage classification using the presented likelihood function is shown in Fig. S1. The correlation of these top 5 diagnostic features (LASSO method) with two cancer groups (early stage and advanced stage) is further demonstrated using hierarchical clustering in a heatmap in Fig. 4. The numerical values of plotted radiomic features are standardized with mean 0 and standard deviation 1. The features are represented on x-axis and their hierarchical clustering is represented on y-axis. Two distinct clusters were formed showing strong association of CP and LDHGLE with the defined cancer groups. However, the heatmap could not fully capture the association of other features with two cancer stages besides CP and LDHGLE.

Finally, in order to integrate cancer and its stage detection equations into one likelihood function, highly diagnostic features namely Surface volume ratio (SVR) and Sum entropy (SE) were chosen as the selection criterion [16]. It was observed through an empirical study carried out on the training data that values of SVR ($z_{1k}$) and SE ($z_{2k}$) are always above threshold values $T_{a}$ and $T_{b}$ respectively for malignant nodules and therefore can be used to estimate likelihood of early and advanced stage cancer from Eq. (9). On the contrary, the values of $z_{1k}$ and $z_{2k}$ below the threshold $T_{a}$ and $T_{b}$ respectively indicate that nodule is benign/non-malignant and its likelihood can be computed using Eq. (8) Using the above mentioned criterion and Eq. (8) and Eq. (9), the proposed likelihood function for multi-class classification is given as follows:

\begin{align*} p(Z_{k} \mid u_{k})= \begin{cases} p_{2}(Z_{k} \mid u_{k}),& \text {if } z_{1k} < T_{a} \text {AND }z_{2k} < T_{b}\\ OR\\ p_{1}(Z_{k} \mid u_{k}), & \text {if } z_{1k}\geq T_{a} \text {AND }z_{2k}\geq T_{b} \end{cases} \\\tag{10}\end{align*}

### C. Algorithm

**FIGURE 5.**
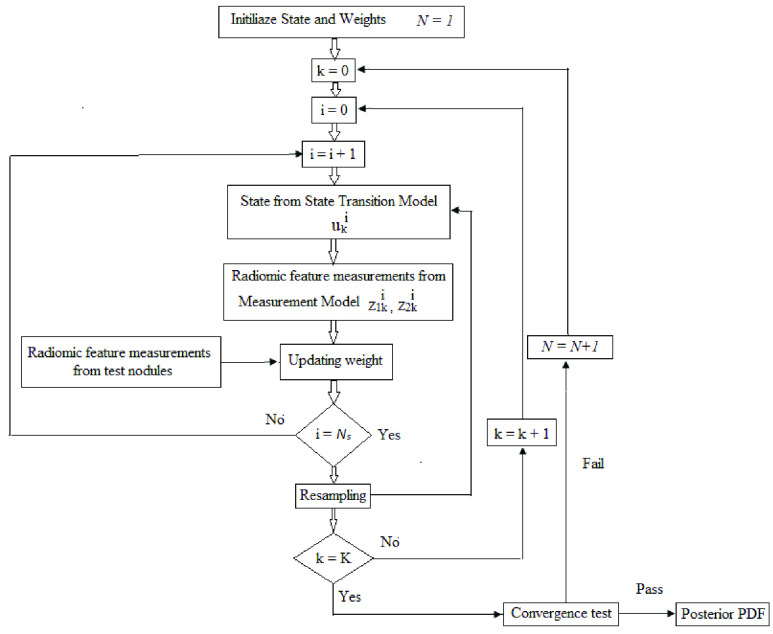
Work flow of the radiomics based Bayesian inversion method for multi-class classification of lung nodules.

The sequence of steps carried out for the proposed diagnostic framework implementation is demonstrated in Fig. 5 and is summarized as follows:

1) To start, $N_{s}$ samples are generated from the prior/state transition PDF in Eq. (7) for the first time screening of a lung nodule. The value of $N_{s}$ is equal to 4000.
2) Next, the features are computed using the measurement function for the known malignancy state from Step 1. For this, an $M^{th}$ order polynomial is proposed as a measurement function as follows:
  \begin{equation*} z_{qk}= \sum _{m=0}^{M}p_{m} u_{k}^{m}\tag{11}\end{equation*}
  Here $z_{qk}$ represents $q^{th}$ diagnostic radiomic feature at $k^{th}$ chest screening. The coefficients $p_{m}$ of polynomial were determined from a training database of known states and the corresponding diagnostic feature measurements. As discussed, two features (SVR($z_{1k}$), SE($z_{2k}$)) were required to estimate benign /malignant state of a nodule [16] and their measurement functions were formulated as:
  \begin{align*} z_{1k}=&\sum _{m=0}^{M}p_{1m} u_{k}^{m} \tag{12}\\ z_{2k}=&\sum _{m=0}^{M}p_{2m} u_{k}^{m}\tag{13}\end{align*}
  The coefficients $p_{1m}$ and $p_{2m}$ were computed for $z_{1k}$ and $z_{2k}$ respectively. Similarly, measurement functions of cancer stage estimation were also developed.
3) Using the features computed in Step 2 and the test radiomics features, two likelihood PDFs were estimated from Eq. (10). The closeness of the two likelihood values obtained determines the sample weight. Large difference refers to small weight assignment and vice versa. Subsequently, the weights were updated using association in Eq. (6).
4) The problem of degeneracy with particles is addressed by performing resampling [30]. After resampling, the posterior PDF of malignancy occurrence was estimated using samples and their associated weights.
5) The PDF estimation process outlined above was repeated for all the cancer screening times from 1 up till $K$.
6) The expectation of state computed from the PDF estimates at $k$ was represented as posterior mean estimate (PME) as follows:
  \begin{equation*} u_{k}^{PME}=E[u_{k} \mid Z_{k}]=\int u_{k} p(u_{k}\mid Z_{k})du_{k}\tag{14}\end{equation*}
7) If the error in single point PDF estimates in two consecutive iterations was within a preset threshold $\xi $, each iteration was denoted by $N$ and the estimated malignancy state of a nodule ($K$ screening times) evaluated at $N$ was $\mathit {u_{1:K}^{N}}$, then the convergence criterion was given by:
  \begin{equation*} \frac {\sum _{k=1}^{K}(u_{k} (N+1)-u_{k} (N))^{2} }{K}\leq \xi\tag{15}\end{equation*}

## V. Data Sets Summary

The proposed particle filtering approach was validated using 200 low dose CT data sets collected from NLST database. From these, there are 130 longitudinal datasets suffering from cancer stage I, II, III or IV respectively at the third year (T3) of screening. Other 70 datasets include patients’ longitudinal CT data diagnosed with benign nodules at the third year (T3) of screening. The dimensions of the acquired images were 512 x 512 pixels. A summary of the test nodules is reported in Table SIII.

## VI. Results

The 3-D segmentation of nodules in lung CT volumes was carried out using Grow Cut algorithm after the verification from a senior radiologist. Pyradomics [4] was used for the extraction of 3-D radiomic feature extraction. The diagnosis results of the filter were evaluated by setting the value of $M$ as 2 and 3 respectively in measurement functions, described in subsection IV-C, Step 2.

With the order of measurement equations set to 2 ($M =2$), the particle filter diagnosed 65 out of 70 benign tumors successfully. In malignant cases, the proposed framework diagnosed 51 out of 70 nodules having early stage cancer and 37 out of 60 nodules with advanced stage cancer correctly. For a comparison, the performance of the proposed framework was assessed using measurement equations of order 3 ($M=3$). A total of 63 out of 70 benign tumors were successfully detected whereas in malignant cases, there were 60 out of 70 nodules with early stage cancer and 49 out of 60 nodules with advanced cancer stage which were correctly diagnosed. Increasing the order $M$ to 4 did not show any significant improvement in the detection results and are excluded in the discussion.

**FIGURE 6.**
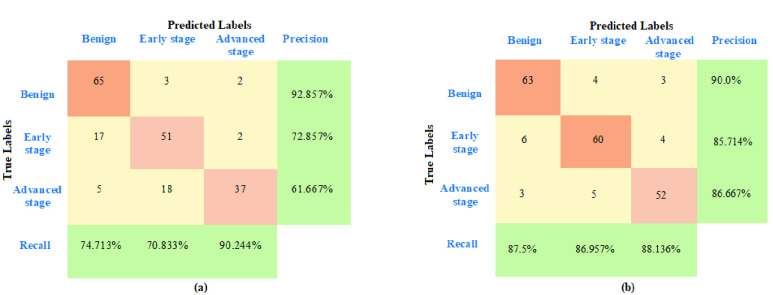
Confusion matrix of cancer detection and stage detection using (a) $M=2$ and (b) $M=3$.

The described diagnostic performance of the filter is expressed in terms of precision and recall for 95% confidence interval along with confusion matrix in Fig. 6. The recall values determine the sensitivity of classification model and precision values compute the positive predictive value of model. The average precision of the filter decreased from 92.86% to 90.0% for benign nodule classification but increased from 72.857% to 85.714% and 61.667% to 86.667% respectively for early and advanced stage cancer estimation when $M$ value was incremented from 2 to 3. The recall metric improved significantly for both benign as well as early stage classification with $M=3$. The overall accuracy of the filter is 86%.

**FIGURE 7.**
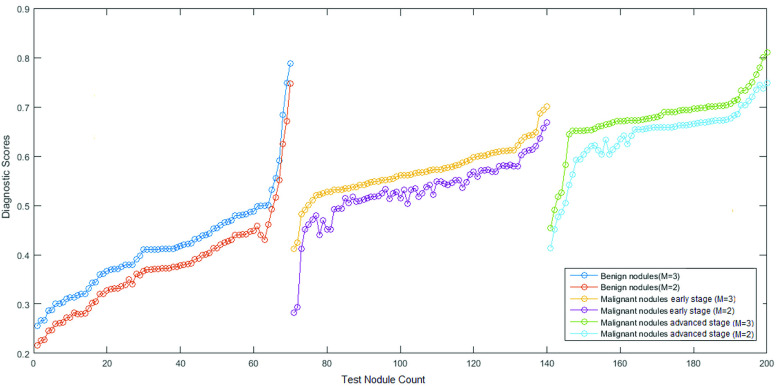
Estimated diagnostic scores of test nodules versus their malignancy status for $M=3$ and $M=2$.

**FIGURE 8.**
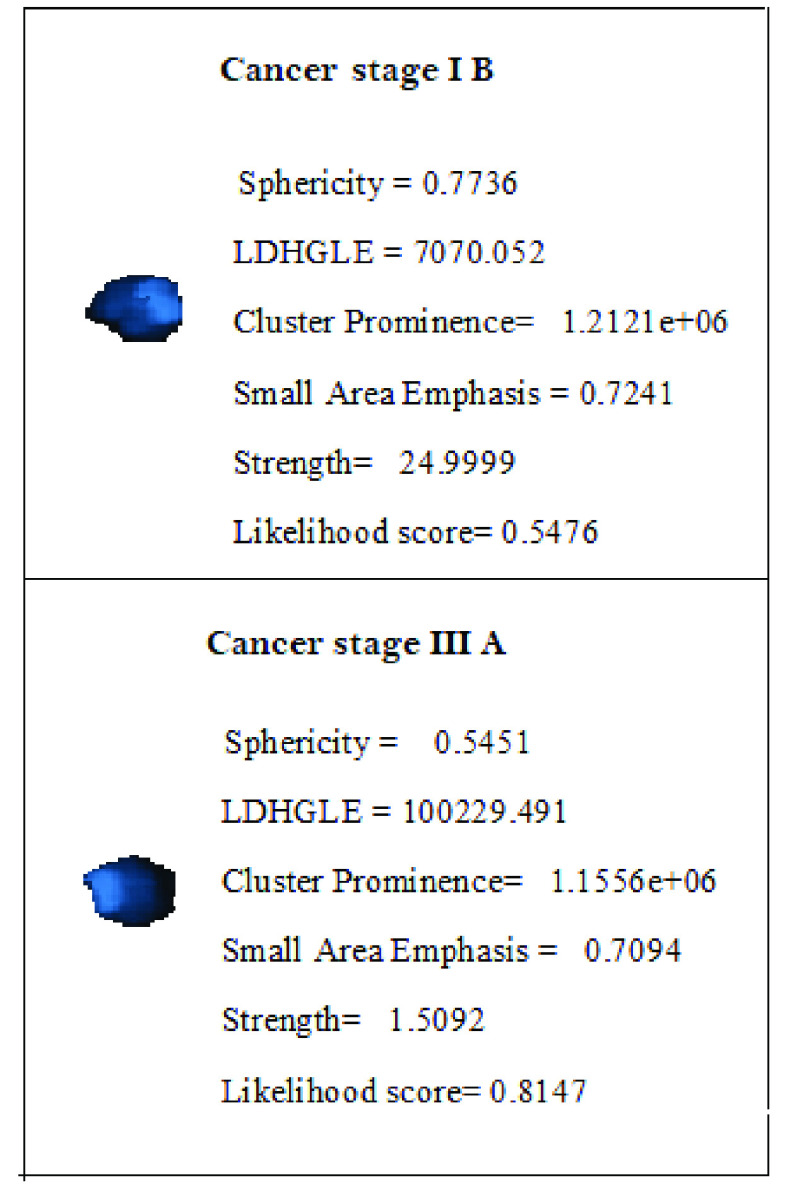
Diagnostic radiomic features and likelihood scores of two sample nodules having cancer stage I B and stage III A.

The estimated diagnostic scores of benign as well as malignant test nodules at final CT screening (k = 5) which is the $4^{th}$ half yearly reading are plotted against each nodule count for $M = 2\,\, \& $ 3 in Fig. 7. Through visual inspection, an overall improvement in the state estimation of malignant lung nodules can be seen using measurement functions of order 3 in PF. To summarize, the diagnostic scores using $M=2$ showed a tendency to shift towards the lower diagnostic ranges causing a spill over in the adjacent classification group. For example, 5 more nodules with early stage cancer obtained a score below 0.51 with $M=2$, hence were diagnosed as benign nodules. Similar trend was observed in nodules with advanced stage cancer where 9 more nodules got diagnosed having early stage cancer stage due to low scores for $M=2$. The above discussed trend in state estimation is illustrated by plotting the state evolution (diagnostic scores) of 1 benign, 1 malignant (cancer stage IB) and 1 malignant (cancer stage IIIA) against the half-yearly CT screenings in Fig. S2. Furthermore, the ROC curves demonstrating the performance of proposed filter for classification of benign, early stage and advanced stage cancer are shown in Fig. S3. The AUCs achieved for benign nodule, early stage cancer and advanced stage cancer detection are 0.8962, 0.8849 and 0.8691 respectively. Computed values of diagnostic features and likelihood scores for stage I B(early stage) and stage III A(advanced stage) cancer are shown in Fig. 8.

## VII. Discussion

The binary classification performance of proposed model into benign and malignant nodules is compared with two other significant models tested on NLST database in Table SIV. The research work in [31] investigated the performance of different classifiers to predict malignancy in screen detected cancer at first year (T0) and second year (T1) of Lung screening trial. A total of 208 nodules were detected among which 104 were diagnosed with cancer. An accuracy (ACC) of 80%, specificity (SPS) of 91% and $AUC =0.83$ were obtained. Authors in [6] analyzed deep learning algorithm generated heat-maps to predict the malignancy in nodules. The presented explainable AI approach to detect malignancy achieved a weighted accuracy of 85%. In comparison, the proposed PF achieved an accuracy of 92.0%, sensitivity of 87.50% and specificity of 94.5% respectively showing excellent performance of the filter in binary classification.

Since no multi-class classification techniques have been reported on NLST datasets in the literature, multi-class classification was performed using SVM with Radial Basis Function (RBF) kernel, Random Forest and K nearest neighbors KNN) classifiers for a comparative performance analysis. The data sets were chosen from third year (T2) screening with 180 datasets used for training and the remaining 20 datasets were chosen for the testing of classifiers. The number of benign and malignant nodules in the experiments were same as used in the proposed filter and reported in Table SIII. The classifier was trained and tested 10 times with 10-fold cross validation. The evaluation metric included averages (for 10 iterations) of precision, recall, accuracy and Mathew’s Correlation Coefficient (MCC) and is reported in Table 2. Classification of nodules as benign, with early stage cancer and advanced stage cancer using SVM, KNN and Random Forest classifiers achieved average precision of 79.08%, 77.20% and 82.79%, recall of 79.09%, 78.25% and 83.23% and an accuracy of 79%, 78% and 83% respectively. In comparison, the proposed particle filter resulted in an average precision of 85.79%, recall of 86.11% and an accuracy of 86% respectively. Furthermore, MCC [32] was computed which achieved values of 71.12%, 70.51%, 76.44% and 80.08% for SVM, KNN, Random Forest and PF respectively. Evidently, the achieved multi-class diagnosis results using diagnostic radiomic features and particle filter are better when compared with the trained SVM, KNN and Random Forest classifier on low dose datasets. The proposed filter has classified 86% of the nodules correctly with fewer input longitudinal data points proving particle filter a good choice for multi-class classification. However, the classification results could be improved with the availability of more chest CT data but is non-attainable due to health hazards attached with multiple CT screenings of a patient. Moreover, a limitation faced to implement the proposed approach is poor management of medical images database currently at many hospitals.

**TABLE 2:** Comparison of Multi-Class Classification Performance Using SVM, Random Forest, KNN and PF

Prediction Model	No. of CT datasets	Prec. %	Recall %	Acc. %	MCC %
SVM	200	79.08	79.09	79.08	71.12
KNN	200	77.20	78.25	78.0	70.51
Random Forest	200	82.79	83.23	83.10	76.44
PF	200	85.79	86.11	86.0	80.08

With proper management of patients’ longitudinal CT data, this research work can be extended to cancer prognostics. Besides current status, the future malignancy status of a nodule can also be estimated using PF approach which re-iterates its efficacy.

## VIII. Conclusion

An accurate and efficient radiomics based Bayesian inversion framework is proposed to detect cancer and its pathological stage as early or advanced stage. This contribution is superior to other prominent cancer detection algorithms whose performances are dependent on large data sets training, a limitation in medical imaging. Moreover, the published contemporary methods do not provide a complete picture of malignancy and its stage using a single research approach as presented in this research work. Furthermore, highly discriminative features towards cancer stages were also identified. The high accuracy of the presented method shows that the proposed model can be used by clinicians in cancer diagnostics.
